# Apropos of Osteopathy

**Published:** 1902-04

**Authors:** 


### Apropos of Osteopathy.

IN A certain type of mind to which the occult appeals with great force is to be found the secret of the perennial vogue of the quack and the charlatan. The physician of today will be entertained and comforted if he will re-read a certain one of Dr. Oliver Wendell Holmes's medical essays in which the rise and fall of various popular delusions of other days are set forth. For the popularity of the latest wonder-cure, osteopathy, the medical profession has itself in no small degree to blame. A therapeutic measure of distinct value in selected cases has been so neglected by the profession generally that a charlatan seizes upon it and seeks to and in some localities succeeds in elevating it into a "school of practice." Surround such a genuine therapeutic measure with a halo of mystery and you have a combination irresistible to the credulous mind. Many physicians are slow to get away from the idea that in drugs alone are found all valuable remedial agents. Medical schools are slow to incorporate in their curricula practical instruction in the use of therapeutic measures outside of materia medica. Mechano-therapy, electricity, hydrotherapy, suggestion, and of not less importance, diet, fresh air, sunlight and exercise, should be remembered as belonging to the armamentarium of the physician, no less than iron, quinine and calomel. Nothing which relieves pain or prolongs life is outside the domain of medicine.

---

The state department of health has announced that it is prepared to furnish diphtheria antitoxin for the treatment and immunisation of persons sick or exposed to diphtheria. A year ago the legislature made an appropriation for the establishment of a laboratory to produce and distribute antitoxins. Last autumn the Bender Hygienic Laboratory, at Albany, began the production of diphtheria antitoxin and the serum is now ready for distribution.

Those entitled to treatment with the state's product are any of the inmates of state or other charitable institutions, hospitals and asylums and any person suffering from or exposed to diphtheria who is unable to purchase this remedy.

---

The health commissioner of Buffalo, Dr. W. D. Greene, lately announced that signs will be placed in all street cars by April 1, 1902, forbidding expectoration in the cars and that, as soon as the signs are in place, the new antispitting ordinance will be enforced to the full extent of the law. The announcement was made as a result of a recent conference between Dr. Greene, Supt. Mitten of the International Traction Company and Supt. Bull of the Police Department.

---

Health Commissioner Greene recently announced the appointment of the five district nurses as sanitary inspectors. These will serve in this capacity without pay, reporting to the health department any violations of sanitary laws which attract their attention. The appointees are: Anna Dean, Alice Meyers, Agnes Odell, Adelaide Colegrove and Anna Damer.

